# A single-center prospective cohort study assessing preoperative gait speed assessment as a prognostic tool for morbidity and mortality in frail elderly patients undergoing abdominal surgery

**DOI:** 10.1590/1414-431X2024e14103

**Published:** 2025-01-31

**Authors:** Ping-Ping Cai, Lu-Lu Gu, Xin Wang, Cui-Li Wu, Xiang-Hong Ye, Kang-Zhen Zhang

**Affiliations:** 1 Jinling Hospital Affiliated Hospital of Medical School, Nanjing University, Nanjing, China; 2 Affiliated Wuxi Fifth Hospital of Jiangnan University, The Fifth People’s Hospital of Wuxi, Wuxi, China; 3 Nanjing Central Hospital, Nanjing, China

**Keywords:** Frailty, Elderly, Abdominal surgery, Complications, Gait speed

## Abstract

Frailty is a significant risk factor for adverse outcomes in elderly surgical patients. Gait speed assessment is a new tool recently used to stratify risk for these pre-operative adverse outcomes. In this prospective study of 392 frail elderly patients undergoing abdominal surgery, we investigated the predictive value of preoperative gait speed for postoperative outcomes. Patients were divided into two groups based on their 6-meter gait speed: normal (≥0.8 m/s, n=184) and slow (<0.8 m/s, n=208). The slow group was older, had more comorbidities, and higher American Society of Anesthesiologists (ASA) grades (P<0.05). They also had significantly higher rates of 30-day overall complications (38.9 *vs* 18.5%, P<0.01), severe complications (12.0 *vs* 4.3%, P<0.01), and 1-year mortality (15.4 *vs* 6.5%, P=0.008) compared to the normal group. Pulmonary infection, wound infection, and delirium were the most common complications. Multivariate logistic regression confirmed slow gait speed as an independent risk factor for 30-day complications (OR=2.38, 95%CI: 1.41-4.01) and 1-year mortality (OR=2.19, 95%CI: 1.07-4.48). Our findings demonstrated that preoperative 6-meter gait speed effectively predicted short-term complications and mid-term mortality in frail elderly patients undergoing abdominal surgery. This suggests the need for individualized perioperative management strategies for high-risk patients with slow gait speed to potentially improve their prognosis.

## Introduction

Population aging is a global trend. People over 65 are the fastest growing age group in both developing and developed countries (1). With the continuous advancement of surgical techniques, the number of elderly patients undergoing surgical treatment is also increasing over time. However, elderly patients have reduced physiological function reserves, weakened stress response capabilities, and often have multiple chronic diseases. Therefore, the incidence of postoperative complications and mortality is significantly higher in the elderly population than in young and middle-aged patients (2). Studies suggest that patients over 65 undergoing elective surgery experience complications 25-30% of the time, and the 30-day postoperative mortality rate is 5-10%; these rates are 3-5 times higher than those seen in young and middle-aged patients (3,4).

Interestingly, frailty is an age-related physiological vulnerability syndrome, which manifests as a decrease in physiological function reserves and a disruption of homeostasis in multiple systems in the body. This results in a reduced ability of the body to respond to internal and external environmental stressors (5). Frailty is recognized as an independent risk factor in elderly surgical patients and is closely related to postoperative complications, prolonged hospital stay, decreased quality of life, and increased mortality (6,7). Therefore, it is crucial to quickly and accurately assess frailty status and surgical risk in elderly patients before surgery to develop reasonable surgical strategies and peri-operative management measures (8).

Many tools have recently been developed to assess frailty (8), including the widely used Fried phenotype (9) and the Clinical Frailty Scale (CFS) (10). However, these methods can be complex to perform and time-consuming, making them difficult to implement widely in clinical practice. Gait speed testing, in comparison, can be used as an objective indicator, reflecting the comprehensive function of multiple systems, such as lower limb muscle strength, balance, coordination, and endurance. This test has the advantages of simplicity, speed, non-invasiveness, and repeatability. It is recommended by domestic and international guidelines as an ideal tool for screening frailty (11,12). Many studies have indicated that gait speed is closely related to the risk of falls, self-care ability, cognitive function, and incidence of cardiovascular events in the elderly, and has been hailed as a “vital sign” for the human body (13). The application of gait speed in preoperative risk assessment of elderly surgical patients has received increasing attention over the recent 10-15 years. Gait speed is now part of the “sarcopenia syndrome,” which is different from “frailty syndrome” (14).

Previous studies using gait speed as a prediction of prognosis in elderly surgical patients focused on cardiac surgery, with few studies on non-cardiac surgery, especially in the field of general surgery. Additionally, there is a lack of prospective cohort studies in China to explore the predictive value of gait speed for adverse outcomes in frail elderly patients undergoing abdominal surgery. Therefore, we aimed to conduct a single-center prospective cohort study to evaluate the predictive ability of using the 6-meter gait speed test in patients to assess 30-day postoperative complication rate and 1-year mortality in frail elderly patients undergoing abdominal surgery. We hoped to provide evidence for surgical risk assessment and clinical decision-making in elderly patients undergoing abdominal surgery.

## Material and Methods

### Study population

Frail elderly patients aged 65 years and older who underwent elective abdominal surgery (gastrointestinal, hepatobiliary, pancreatic, colorectal, etc.) in Jinling Hospital Affiliated Hospital of Medical School, Nanjing University from December 2018 to December 2023 were prospectively and consecutively enrolled. The inclusion criteria were: 1) age ≥65 years; 2) planned to undergo open or laparoscopic surgery under general anesthesia; 3) preoperative assessment met the criteria for frailty (Fried phenotype score ≥3 or CFS score ≥5) (3,4); 4) no severe cognitive impairment and cooperative with gait speed testing; and 5) voluntarily signed informed consent. The exclusion criteria were: 1) underwent cardiopulmonary resuscitation before surgery; 2) traumatic surgery; and 3) had neuromuscular diseases affecting lower limb motor function. The hospital ethics committee approved this study. All patients or their legal guardians signed a written informed consent form before enrollment, and the privacy and rights of the patients were fully protected during the research process. This study was conducted and reported in accordance with the Strengthening the Reporting of Observational Studies in Epidemiology (STROBE) guidelines for observational studies.

### Data collection

We collected the following preoperative variables: age, gender, height, weight, education level, smoking history, drinking history, chronic disease history (hypertension, diabetes, chronic obstructive pulmonary disease), American Society of Anesthesiologists (ASA) classification, Charlson Comorbidity Index (CCI) score, and preoperative laboratory tests (routine blood work, biochemistry, coagulation, electrocardiogram, chest radiograph, and abdominal imaging). We collected the following intraoperative data: type of surgery (open or laparoscopic), duration of surgery, estimated blood loss, type of anesthesia, and surgical site classification (clean, clean-contaminated, contaminated). The following postoperative complications were classified according to the Clavien-Dindo classification: wound infection, pulmonary infection, urinary tract infection, anastomotic leakage, ileus, bleeding, delirium, and cardiovascular events.

### 6-meter gait speed test

To perform the 6-meter gait speed test, starting and ending points were marked on the ground of a flat and well-lit corridor. Patients were instructed to stand at the starting point and walk straight to the end point at a natural and comfortable pace without the assistance of others or assistive devices. Acceleration and deceleration zones, located at 2 m before and after the starting point, were not included in the test distance. We used a stopwatch to record the time each subject took to walk the 6-m distance, and the gait speed (m/s) was obtained by dividing the distance by the time. The average of three tests was used as the subject’s score. Patients were divided into a normal group (≥0.8 m/s) and a slow group (<0.8 m/s) according to their gait speed (15,16).

### Surgical methods and postoperative management

All surgeries were performed by experienced senior attending physicians. Open or laparoscopic surgery was selected according to the patient's condition, and all were performed under general anesthesia with endotracheal intubation. The principles of intraoperative fluid infusion, blood transfusion, and analgesia were consistent. Routine antibiotics were given postoperatively to prevent infection, and patients were encouraged to ambulate early. Nutritional support and complication management were provided when necessary.

### Outcome measures

We assessed complications within 30 days after surgery according to the Clavien-Dindo classification criteria (5) and recorded various complications that occurred within 30 days after surgery. Complications were classified as mild (grade I-II) and severe (≥grade III). We calculated the overall incidence of complications and the incidence of individual complications (wound infection, pulmonary infection, delirium, etc.). We also assessed 1-year mortality by following all patients for 1 year; deaths from any cause were recorded. We then calculated the cumulative 1-year mortality rate.

### Statistical analysis

We used SPSS 23.0 statistical software (IBM, USA) for data analysis. Data are reported as means±SD and were compared between groups using *t*-tests. Count data are reported as number and percentage and were compared between groups using the chi-squared test or the Fisher's exact probability method. We used multivariate logistic regression to analyze the relationship between gait speed, complications, and mortality, and we calculated odds ratios (ORs) and 95% confidence intervals (CIs). A P value <0.05 was considered to be statistically significant.

## Results

### General information

A total of 392 patients were included in the study, with 184 in the normal gait speed group and 208 in the slow gait speed group. The slow gait speed group was significantly older, and had a higher proportion of females, lower education levels, higher Fried phenotype and CFS scores, higher CCI scores, as well as a higher proportion of ASA grade III-IV (all P<0.05; Table 1).

**Table 1:** Patient characteristics.

	Normal gait speed (n=184)	Slow gait speed (n=208)	P value
Age (years)	73.3±5.7	75.8±6.2	<0.05
Gender			<0.05
Men (n)	106 (57.6%)	95 (45.7%)	
Women (n)	78 (42.4%)	113 (54.3%)	
Educational level			<0.01
Primary school and below	83 (45.1%)	129 (62.0%)	
Middle school and above	101 (54.9%)	79 (38%)	
Body mass index (kg/m^2^)	23.2±3.4	22.8±3.7	>0.05
Smoking history	62 (33.7%)	79 (38.0%)	>0.05
Drinking history	45 (24.5%)	58 (27.9%)	>0.05
Comorbid conditions			
Hypertension	96 (52.2%)	137 (65.9%)	<0.01
Diabetes	43 (23.4%)	71 (34.1%)	<0.05
COPD	28 (15.2%)	52 (25.0%)	<0.05
Fried phenotype	2.4±0.6	3.8±0.8	<0.01
CFS scores	4.2±0.5	5.5±0.7	<0.01
CCI scores	2.1±1.2	2.8±1.4	<0.01
ASA grade			<0.01
I-II level	128 (69.6%)	108 (51.9%)	
III-IV level	56 (30.4%)	100 (48.1%)	
Surgical site			>0.05
Gastrointestinal tract	72 (39.1%)	89 (42.8%)	
Liver, bile tract, pancreas	63 (34.2%)	75 (36.1%)	
Colon and rectum	49 (26.6%)	44 (21.2%)	

Data are reported as means and SD or number and percentage. Groups were compared with Student’s *t*-test or chi-squared test. COPD: chronic obstructive pulmonary disease; CFS: Clinical Frailty Scale; CCI: Charlson Comorbidity Index; ASA: American Society of Anesthesiologists.

### Relationship between gait speed and complications within 30 days after surgery

The slow gait speed group had a significantly higher overall complication rate (38.9 *vs* 18.5%, P<0.001), as well as higher rates of both mild and severe complications (P<0.01). Pulmonary infection, wound infection, and delirium were the main complications, all occurring more frequently in the slow gait speed group (P<0.05; Table 2).

**Table 2:** Incidence of complications within 30 days after surgery between groups [n (%)].

Complication type	Normal walking speed group (n=184)	Slow walking speed group (n=208)	X^2^ value	P value
Total complications	34 (18.5)	81 (38.9)	19.175	<0.001
Mild complications	26 (14.1)	56 (26.9)	9.328	0.002
Serious complications	8 (4.3)	25 (12.0)	7.468	0.006
Pulmonary infection	14 (7.6)	36 (17.3)	8.212	0.004
Incision infection	7 (3.8)	20 (9.6)	5.183	0.023
Delirium	4 (2.2)	15 (7.2)	5.518	0.019

### Multivariate analysis of gait speed and postoperative complications

After adjusting for confounding factors, multivariate logistic regression analysis indicated that a gait speed of <0.8 m/s was an independent risk factor for complications within 30 days after surgery (OR=2.38, 95%CI: 1.41-4.01, P=0.001).

### Relationship between gait speed and 1-year mortality

**Figure 1:**
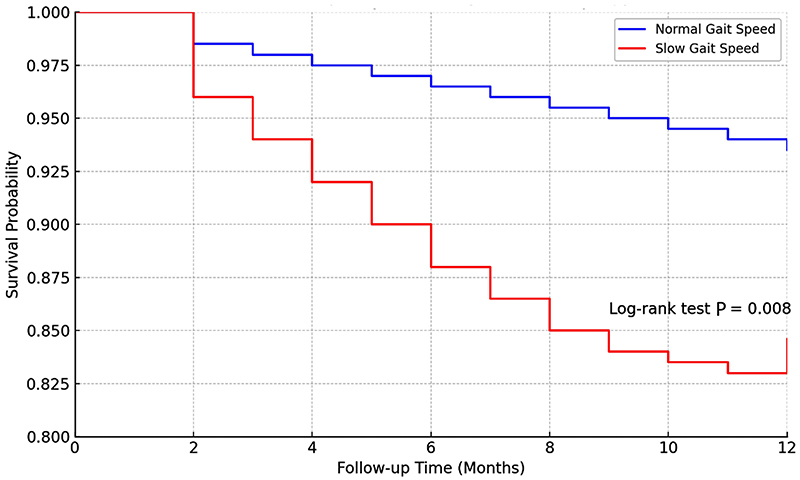
Kaplan-Meier survival curve comparing 1-year mortality between normal and slow gait speed groups.

In total, 380 patients (96.9%) had the 1-year follow-up completed. The slow gait speed group had a significantly higher 1-year mortality rate compared to the normal gait speed group (15.4 *vs* 6.5%, P=0.008). Multivariate analysis confirmed that a gait speed of <0.8 m/s was an independent risk factor for 1-year mortality (OR=2.19, 95%CI: 1.07-4.48, P=0.032) (Figure 1).

## Discussion

This prospective cohort study demonstrated that for frail elderly patients aged 65 years and older undergoing elective abdominal surgery, a preoperative 6-meter gait speed <0.8 m/s was associated with higher 30-day complication rates and 1-year mortality. Slow gait speed may be an independent risk factor for both short-term complications and mid-term mortality, underscoring its value in predicting surgical outcomes.

All patients included in this study were frail elderly patients undergoing abdominal surgery, with the Fried phenotype and CFS scores used to screen the frail population. Interestingly, the overall incidence of complications (38.9%) and mortality (15.4%) were significantly higher than those reported in previous studies. Literature suggests that the incidence of complications in patients undergoing elective general surgery is 17-29%, and the 30-day postoperative mortality rate is 1.2-5.7% (17,18). Therefore, these higher rates of complications and mortality in our study population underscore the increased vulnerability of frail elderly patients undergoing abdominal surgery. This marked difference highlights the critical need for specialized preoperative assessment and tailored perioperative care strategies for this high-risk group. It also emphasizes the importance of using frailty screening tools, such as the Fried phenotype and CFS scores, to identify patients at elevated risk of adverse outcomes. These findings suggested that current standard preoperative risk assessments may underestimate the actual risk in frail elderly patients, pointing to the need for more targeted interventions and potentially more conservative approaches to elective surgeries in this population.

Our work can be compared with the recently published Peri-interventional Outcome Study in the Elderly (POSE) (19), which includes a large-scale European prospective cohort study of 9,497 patients aged 80 and older undergoing various procedures. The POSE study reported a 30-day mortality rate of 4.2%, which is lower than our observed 1-year mortality of 15.4%. Despite population differences, both studies identified that frailty is a significant risk factor for mortality.

Furthermore, multiple studies indicate that preoperative gait speed is associated with adverse postoperative outcomes. Sultan et al. (20) reported that for every 25-m decrease in preoperative 6-minute walking distance in cardiac surgery patients, the risk of postoperative complications increased by 6% (OR=1.06, 95%CI: 1.03-1.09). Additionally, Karlsson et al. (21) reported that patients over 80 years of age with a preoperative gait speed of <0.6 m/s undergoing hip fracture surgery had significantly higher 90-day mortality and 1-year disability rates. Fagard et al. (22) found that in colorectal cancer patients aged 65 years and older, a preoperative gait speed of <1 m/s was associated with increased incidence of severe complications after surgery.

Our study focused on frail elderly patients and used 0.8 m/s as the gait speed cut-off point, confirming that slow gait speed is an independent risk factor for overall complications (OR=2.38) and severe complications (OR=2.78) within 30 days after abdominal surgery. Additionally, the 1-year cumulative mortality rate in frail elderly patients with slow gait speed was 15.4%, 2.37 times higher than those with normal gait speed. These findings support much of the previous literature (16,23) that suggests that gait speed is associated with survival in elderly populations and chronic obstructive pulmonary disease patients.

Although testing methods vary across studies, most utilize 0.8-1.0 m/s as the cut-off value for gait speed in the elderly population (24,25). This range is clinically significant as it often distinguishes between those with normal and slow gait speeds, with slower speeds being associated with increased risk of adverse health outcomes, decreased functional independence, and potentially poorer surgical outcomes in elderly patients. Our study adopted a lower threshold of 0.8 m/s, considering the frail nature of our subjects and the impact of abdominal surgery on the fragility of the patients. Even with this lower threshold, 53.1% of patients had abnormal gait speed, much higher than 20-30% in the general elderly population (26).

Moreover, for high-risk frail elderly patients with slow gait speed before surgery, we recommend individualized management strategies, including systematic evaluation of comorbidities, minimally invasive surgery when possible, enhanced postoperative monitoring, and extended follow-up focusing on long-term quality of life.

The limitations of this study include its single-center design with a relatively small sample size, potential selection bias due to the inclusion of various abdominal surgeries, lack of a control group for comparing frail and non-frail patients, and a limited 1-year follow-up period. To address these issues, future research should focus on conducting multi-center, large-scale prospective cohort studies with longer follow-up periods. These studies should include a matched non-frail control group, allowing for comparison of gait speed's predictive ability across different populations. Additionally, further optimization of the gait speed stratification scheme and development of a comprehensive risk prediction model incorporating gait speed is needed. Such research will enhance our understanding of the ability for assessing gait speed to aid in prognosis in elderly surgical patients and contribute to more effective peri-operative management strategies.

## Conclusion

Our study underscored the importance of a comprehensive geriatric assessment, including the evaluation of gait speed in elderly surgical patients. This simple, objective measure provides valuable insight into patient risk and can guide peri-operative management strategies to improve outcomes in this vulnerable population.

## References

[1] Hamidi M, Joseph B (2019). Changing epidemiology of the American population. Clin Geriatr Med.

[2] Soresina A, Moratto D, Chiarini M, Paolillo C, Baresi G, Focè E (2020). Two X‐linked agammaglobulinemia patients develop pneumonia as COVID‐19 manifestation but recover. Pediatri Allergy Immunol.

[3] Seib CD, Rochefort H, Chomsky-Higgins K, Gosnell JE, Suh I, Shen WT (2018). Association of patient frailty with increased morbidity after common ambulatory general surgery operations. JAMA Surg.

[4] Bollegala N, Jackson TD, Nguyen GC (2016). Increased postoperative mortality and complications among elderly patients with inflammatory bowel diseases: an analysis of the national surgical quality improvement program cohort. Clin Gastroenterol Hepatol.

[5] Fried LP, Tangen CM, Walston J, Newman AB, Hirsch C, Gottdiener J (2001). Frailty in older adults: evidence for a phenotype. J Gerontol A Biol Sci Med Sci.

[6] Vermeiren S, Vella-Azzopardi R, Beckwee D, Habbig AK, Scafoglieri A, Jansen B (2016). Frailty and the prediction of negative health outcomes: a meta-analysis. J Am Med Dir Assoc.

[7] Lin HS, Watts JN, Peel NM, Hubbard RE (2016). Frailty and post-operative outcomes in older surgical patients: a systematic review. BMC Geriatr.

[8] Hernández-Aguiar Y, Becerra-Bolaãos A, Rodríguez-Pérez A (2024). Preoperative diagnosis of frailty. J Int Med Res.

[9] Chen S, Chen T, Kishimoto H, Susaki Y, Kumagai S (2020). Development of a fried frailty phenotype questionnaire for use in screening community-dwelling older adults. J Am Med Dir Assoc.

[10] Church S, Rogers E, Rockwood K, Theou O (2020). A scoping review of the Clinical Frailty Scale. BMC Geriatr.

[11] Afilalo J, Alexander KP, Mack MJ, Maurer MS, Green P, Allen LA (2014). Frailty assessment in the cardiovascular care of older adults. J Am Coll Cardiol.

[12] Chen X, Mao G, Leng SX (2014). Frailty syndrome: an overview. Clin Interv Aging.

[13] Studenski S (2009). Bradypedia: is gait speed ready for clinical use?. J Nutr Health Aging.

[14] de Salles ICD, Sernik R, da Silva JLP, Taconeli C, Amaral AA, de Brito CMM (2023). Sarcopenia, frailty, and elective surgery outcomes in the elderly: an observational study with 125 patients (the SAFESOE study). Front Med (Lausanne).

[15] Nolan CM, Maddocks M, Maher TM, Canavan JL, Jones SE, Barker RE (2018). Phenotypic characteristics associated with slow gait speed in idiopathic pulmonary fibrosis. Respirology.

[16] Studenski S, Perera S, Patel K, Rosano C, Faulkner K, Inzitari M, Jet al (2011). Gait speed and survival in older adults. JAMA.

[17] Stabenau HF, Becher RD, Gahbauer EA, Leo-Summers L, Allore HG, Gill TM (2018). Functional trajectories before and after major surgery in older adults. Ann Surg.

[18] Berian JR, Mohanty S, Ko CY, Rosenthal RA, Robinson TN (2016). Association of loss of independence with readmission and death after discharge in older patients after surgical procedures. JAMA Surg.

[19] POSE-Stydy Group (2022). Peri-interventional outcome study in the elderly in Europe: a 30-day prospective cohort study. Eur J Anaesthesiol.

[20] Sultan P, Hamilton MA, Ackland GL (2012). Preoperative muscle weakness as defined by handgrip strength and postoperative outcomes: a systematic review. BMC Anesthesiol.

[21] Karlsson A, Berggren M, Gustafson Y, Olofsson B, Lindelöf N, Stenvall M (2016). Effects of geriatric interdisciplinary home rehabilitation on walking ability and length of hospital stay after hip fracture: a randomized controlled trial. J Am Med Dir Assoc.

[22] Fagard K, Casaer J, Wolthuis A, Flamaing J, Milisen K, Lobelle JP (2017). Postoperative complications in individuals aged 70 and over undergoing elective surgery for colorectal cancer. Colorectal Dis.

[23] Nakano T, Kimura S, Yamashita T, Yoshimi M, Tao Y, Takata S (2021). Correlation of 4-meter gait speed with clinical indicators of chronic obstructive pulmonary disease. Respir Investig.

[24] Peel NM, Kuys SS, Klein K (2013). Gait speed as a measure in geriatric assessment in clinical settings: a systematic review. J Gerontol A Biol Sci Med Sci.

[25] Pamoukdjian F, Paillaud E, Zelek L, Laurent M, Lévy V, Landre T (2015). Measurement of gait speed in older adults to identify complications associated with frailty: a systematic review. J Geriatr Oncol.

[26] van Kan GA, Rolland Y, Andrieu S, Bauer J, Beauchet O, Bonnefoy Ml (2009). Gait speed at usual pace as a predictor of adverse outcomes in community-dwelling older people an International Academy on Nutrition and Aging (IANA) Task Force. J Nutr Health Aging.

